# Non-linear associations between UHR and bone mineral density in US adults: NHANES 2017–2018

**DOI:** 10.1186/s40001-025-03200-3

**Published:** 2025-11-08

**Authors:** Han Wen, Minfeng Zhou, Huifang Niu, Huazhen Xia

**Affiliations:** 1 Hunan University of Medicine, The First People’s Hospital of Pingjiang, 431 North Street, Chengguan Town, Pingjiang County, 414500 China; 2 Union Hospital Affiliated to Tongji Medical College, Huazhong University of Science and Technology, 1277 Liberation Avenue, Wuhan City, 430022 China; 3 Jianghan University School of Medicine, 8 Triangle Lake Road, Wuhan City, 430056 China; 4 Jianghan University Institute of Acupuncture and Moxibustion, 8 Triangle Lake Road, Wuhan City, 430056 China; 5 School of Nursing, Shenyang Medical College, Shenyang, 110034 China

## Abstract

**Introduction:**

Bone mineral density (BMD) is a key indicator of bone health, particularly in older populations, where lower BMD is linked to increased risk of osteoporosis and fractures. Metabolic factors like serum uric acid (UA) and high-density lipoprotein cholesterol (HDL-C) have emerged as possible determinants of bone health. The uric acid to HDL cholesterol ratio (UHR) may offer a new perspective on these metabolic influences. This study explores the association between UHR and femoral neck BMD, with a focus on non-linear relationships and subgroup variations by body mass index (BMI), age, and sex.

**Methods:**

The study used data from 2178 participants from the 2017–2018 National Health and Nutrition Examination Survey (NHANES). UHR was calculated as the ratio of serum UA to HDL-C. BMD measurements were obtained using dual-energy X-ray absorptiometry (DXA) at the femoral neck. A two-piecewise linear regression model was applied to examine the non-linear relationship between UHR and BMD. Stratified analyses were conducted by BMI, gender, and age groups.

**Results:**

A significant inflection point was found at UHR 19. Below this threshold, UHR was positively associated with femoral neck BMD (*β* = 0.0054, *p* = 0.013), while above the threshold, the association was negative but not statistically significant (*β* = − 0.0016,* p* = 0.478). Stratified analysis revealed that the relationship between UHR and BMD remained significant among Mexican Americans even after adjusting for covariates (*β* = 0.0145, *p* = 0.012).

**Conclusion:**

This study identifies a non-linear association between UHR and femoral neck BMD, with a key inflection point at UHR 19. These findings suggest that UHR could be a useful biomarker for bone health, especially in populations with higher metabolic risks. Further longitudinal studies are necessary to establish causality and explore potential interventions targeting UHR to improve bone health.

## Introduction

Bone mineral density (BMD) is crucial in assessing bone health, particularly in older populations where decreases in BMD are closely linked to osteoporosis and fracture risks [1]. Osteoporosis, often referred to as the ‘silent disease,’ progresses without symptoms until a fracture occurs, which makes it a particularly challenging issue for public health [2]. The lack of early warning signs makes diagnosis and prevention more challenging, thereby increasing the health burden of osteoporotic fractures [3]. Globally, osteoporosis continues to pose a major public health challenge due to its contribution to increased morbidity and mortality [4–6]. Recent studies have identified metabolic factors, such as UA and HDL-C, as potential determinants of bone health, further emphasizing the need for early detection and intervention [7–9].

UA, a product of purine metabolism, plays a dual role as both a pro-oxidant and an antioxidant in the body. While elevated UA levels have been traditionally linked to gout, cardiovascular diseases, and metabolic syndrome, emerging evidence suggests that UA may protect against bone loss due to its antioxidant properties [10–12]. For instance, Ishii et al. demonstrated that elevated serum UA levels positively correlated with lumbar spine BMD in peri- and postmenopausal Japanese women [13]. Similarly, Yan et al. and Zhao et al. observed a positive association between UA levels and BMD in Chinese patients with type 2 diabetes mellitus [14, 15]. However, other studies have shown conflicting results, where elevated UA levels were negatively associated with bone health in individuals with metabolic disorders [16].

HDL-C, long recognized for its cardiovascular benefits, is also believed to play a role in bone metabolism. Research by Xie et al. found a significant positive association between HDL-C and BMD in U.S. adults [17]. Several other studies support this, suggesting that lipid metabolism may influence bone density [18–20]. However, a large-scale cohort study conducted by Sultana Monira Hussain et al. identified a significant correlation between HDL-C levels and an elevated risk of fractures [21], highlighting the complex and potentially paradoxical role of HDL-C in bone health.

The uric acid to HDL cholesterol ratio (UHR) has recently been proposed as a potential biomarker reflecting the balance between UA and HDL-C and their combined impact on bone health [22, 23]. Despite growing interest, limited research has focused directly on the relationship between UHR and BMD. Moreover, most studies have focused on non-weight-bearing bones, such as the lumbar spine, while weight-bearing bones, like the femoral neck—more susceptible to metabolic influences—have received less attention [24]. Additionally, the influence of demographic variables such as sex, age, and body mass index (BMI) on the UHR-BMD relationship remains poorly understood.

This study aims to assess the relationship between UHR and femoral neck BMD, with a focus on determining whether a non-linear relationship exists. Additionally, potential variations across gender, age, and BMI subgroups will be explored. Findings from this study may provide valuable insights into metabolic influences on bone health and offer new perspectives for the prevention and treatment of osteoporosis.

## Materials and methods

### Study population

This study utilized health data from all participants in the 2017–2018 NHANES. According to the NHANES website, the survey received approval from the Ethics Review Board (ERB) of the National Health and Health Commission.

### Sample inclusion criteria

**Fig. 1:**
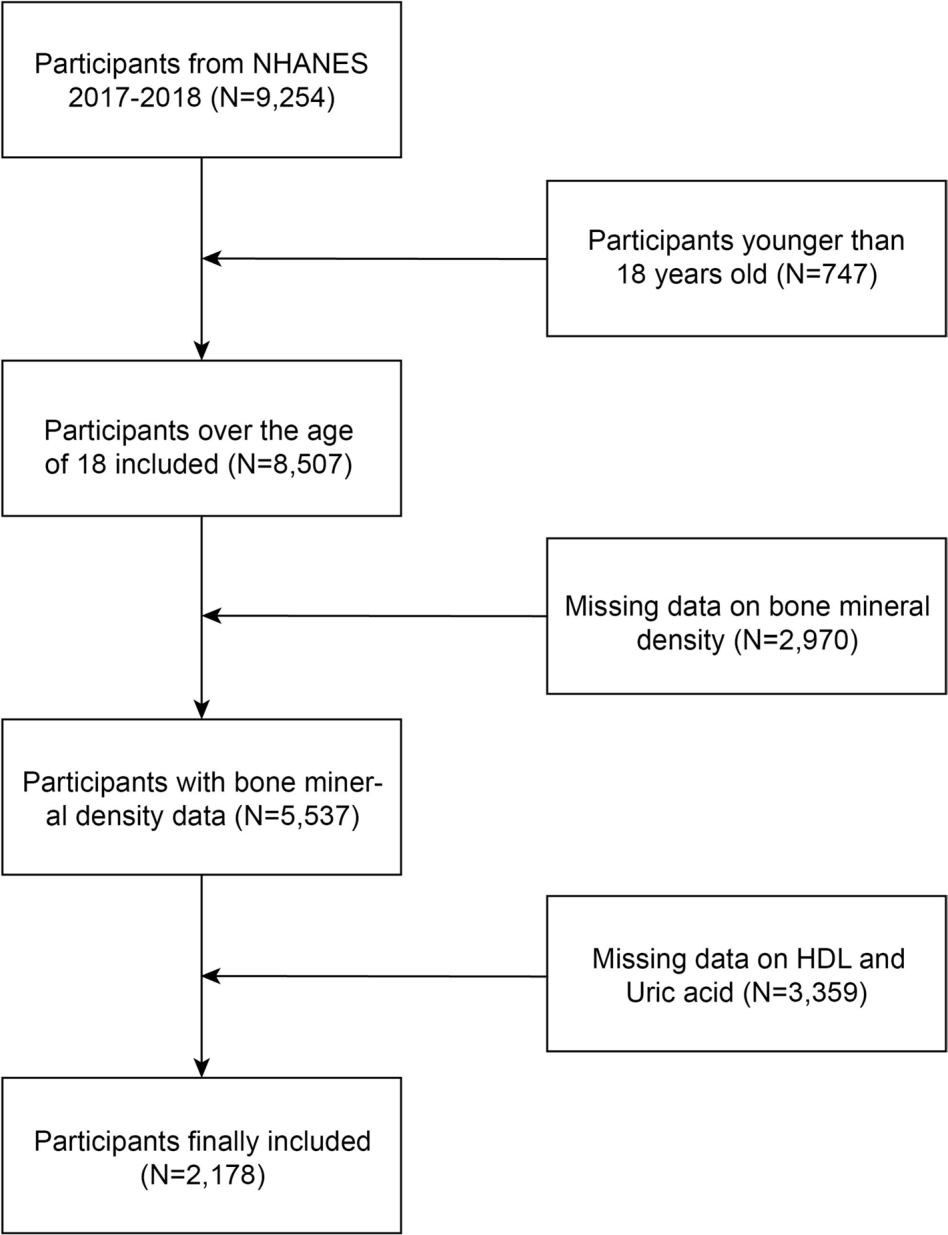
Flowchart of Participant Inclusion from NHANES 2017–2018

From the 9254 participants in the 2017–2018 NHANES cycle, 747 individuals were excluded due to age (< 18 years), and 2970 and 3359 participants were excluded due to missing data on BMD, uric acid, and HDL, respectively. A total of 2178 participants were included in the final analysis, as shown in Fig. 1.

### Definition of variables

Demographic variables were collected using the Computer-Assisted Personal Interview (CAPI) system by trained interviewers at participants’ homes. The variables included age (20–29, 30–44, 45–59, 60–79 years), gender (male, female), race/ethnicity (Mexican American, Other Hispanic, Non-Hispanic White, Non-Hispanic Black, Other Race including multiracial), education level (less than 9th grade, 9–11th grade, high school graduate/GED or equivalent, some college or AA degree, college graduate or above), marital status, and family poverty–income ratio. Physical activity was categorized into vigorous, moderate, and no activity based on daily duration and frequency, and smoking status was classified as current, former, or never smokers based on questionnaire responses. Hypertension was defined based on responses to whether the participant had ever been diagnosed with hypertension, was told by a doctor to take antihypertensive medications, or was currently taking prescription medications for hypertension. Diabetes was defined by responses indicating a doctor’s diagnosis of diabetes, current insulin use, or use of diabetic pills to lower blood sugar. Body measurement data included BMI and femoral neck BMD. The 2017–2018 femur scans were performed using Hologic QDR-4500A fan-beam densitometers (Hologic, Inc., Bedford, Massachusetts) with Apex 3.2 software. The radiation dose from DXA scans was minimal, at less than 20 µSv. All scans in the BMD dataset were analyzed using Hologic APEX version 4.0 software. Trained and certified radiology technologists administered the DXA exams, and further details of the DXA protocol are documented in the Body Composition Procedures Manual available on the NHANES website. Laboratory data included C-reactive protein (CRP), alanine aminotransferase (ALT), calcium, phosphorus, creatinine, urea nitrogen, serum uric acid, and HDL. Detailed information on the laboratory methods, samples, equipment, and addresses can be found in the corresponding module on the NHANES website:

https://www.cdc.gov/nchs/nhanes/index.htm.

### Definition of exposure and outcome variables

UHR was defined as the ratio of serum uric acid to HDL-C, multiplied by 100%, to yield UHR (%). Bone mineral density in this study was measured using femoral neck BMD.

### Statistical analysis

To represent the national population’s health, sample weights were applied. Continuous data were expressed as weighted mean ± standard deviation (SD), and categorical variables were presented as weighted proportions. BMD was divided into tertiles. A weighted Chi-square test was used for categorical variables, while a weighted linear regression model was used for continuous variables to assess group differences. Weighted multivariate linear regression models were employed to study the relationship between UHR and femoral neck BMD, with stratified analyses by gender, age, and race/ethnicity. Three models were adjusted: the first was unadjusted, the second adjusted for gender, age, and race/ethnicity, and the third adjusted for all variables except the exposure and outcome variables. A smoothing curve fitting and generalized additive model were used to determine the non-linear relationship between UHR and femoral neck BMD, and saturation effect analysis was performed based on the results. All analyses were conducted using EmpowerStats and R (http://www.R-project.org). A *p*-value < 0.05 was considered statistically significant.

## Results

### Baseline characteristics of the population

Table 1 highlights significant differences across femoral neck BMD tertiles (T1: < 0.688, T2: 0.689–0.810, T3: ≥ 0.811) in demographic, health, and metabolic factors. Age was lower in T3 (61.11 ± 8.01 years) compared to T1 (65.86 ± 9.12 years) (*p* < 0.001). Males were more prevalent in T3 (68.2%) compared to T1 (26.66%), while females were more common in T1 (*p* < 0.001). Non-Hispanic Whites were more prevalent in T1, whereas Non-Hispanic Blacks were more common in T3 (*p* < 0.001). Diabetes, severe alcohol consumption, and smoking were more frequent in T3 than in T1 (*p* < 0.001, *p* = 0.015). Metabolic markers such as BMI, CRP, ALT, UHR, uric acid, creatinine, and phosphorus levels were higher in T3, while HDL levels were lower (*p* < 0.001). In contrast, no significant differences were found between T1 and T3 in education level, hypertension, physical activity, income-to-poverty ratio, albumin, urea nitrogen, and calcium (*p* > 0.05).

**Table 1:** Weighted characteristics of the study population based on femoral neck bone mineral density (g/cm^2^)

Femoral neck BMD(g/cm^2^,mean ± SD) (*N* = 2178)	T1 < 0.688	T2 (0.689–0.810)	T3 ≥ 0.811	*P*-value
Gender, *n* (%)				< 0.001
Male	26.66	52.53	68.2	
Female	73.34	47.47	31.8	
Age (years)	65.86 ± 9.12	63.07 ± 8.45	61.11 ± 8.01	< 0.001
Race, *n* (%)				< 0.001
Mexican American	3.98	6.27	6.11	
Other Hispanic	5.02	7.56	6.69	
Non-Hispanic White	76.52	69.9	62.28	
Non-Hispanic Black	4.48	8.24	14.91	
Other Race	10	8.02	10.01	
Education level, *n* (%)				0.539
Less than 9th grade	3.58	3.97	4.12	
9–11th grade	7.15	7.9	5.31	
High school grade	30.52	26.8	29.52	
Some college or AA degree	27.37	29.97	29.84	
College graduate or above	31.38	31.35	31.21	
Marital status, *n* (%)				< 0.001
Married	56.87	65.21	64.8	
Widowed	17.98	5.93	5.82	
Divorced	16.94	17.49	13.18	
Separated	1.61	1.53	3.36	
Never married	4.39	4.48	7.61	
Living with partner	2.2	5.37	5.23	
Alcohol use, *n* (%)				< 0.001
Never	10.93	5.83	5.99	
Moderate	61	58.79	56.31	
Severe	28.07	35.38	37.69	
Smoke status, *n* (%)				0.015
No	60.76	54.51	52.93	
Ever	27.3	32.76	31.49	
Current	11.94	12.73	15.58	
Diabetes, *n* (%)				< 0.001
No	87.13	79.75	77.48	
Yes	12.87	20.25	22.52	
Hypertension, *n* (%)				0.064
No	53.89	47.82	51.22	
Yes	46.11	52.18	48.78	
Physcial activity, *n* (%)				< 0.001
Never	54.87	49.08	47.26	
Moderate	9.78	9.94	5.79	
Severe	35.36	40.98	46.94	
Body mass index (kg/m^2^)				< 0.001
< 25	39.86	21.51	8.61	
25–30	34.81	36.92	34.36	
≥ 30	25.32	41.57	57.03	
Income-to-poverty ratio	3.08 ± 1.54	3.22 ± 1.52	3.29 ± 1.54	0.031
CRP (mg/L)	3.39 ± 8.40	4.03 ± 7.35	5.01 ± 10.88	0.003
ALT (U/L)	20.17 ± 11.33	23.01 ± 16.65	23.83 ± 13.92	< 0.001
Albumin (g/dL, mean ± SD)	4.05 ± 0.29	4.05 ± 0.30	4.07 ± 0.28	0.410
Urea Nitrogen (mg/dL, mean ± SD)	16.53 ± 5.49	16.65 ± 5.76	16.73 ± 5.31	0.793
Creatinine (mg/dL, mean ± SD)	0.89 ± 0.36	0.92 ± 0.33	0.95 ± 0.25	0.003
Phosphorus (mg/dL, mean ± SD)	3.65 ± 0.48	3.56 ± 0.52	3.51 ± 0.54	< 0.001
Calcium (mg/dL, mean ± SD)	9.33 ± 0.39	9.34 ± 0.35	9.32 ± 0.35	0.406
Uric acid (mg/dL, mean ± SD)	5.00 ± 1.32	5.60 ± 1.42	5.89 ± 1.38	< 0.001
HDL (mg/dL, mean ± SD)	60.23 ± 15.80	53.06 ± 15.20	51.47 ± 15.23	< 0.001
UHR (%)	9.12 ± 4.03	11.57 ± 4.74	12.55 ± 4.87	< 0.001

Mean ± SD was for continuous variables. The *p*-value was calculated by the weighted linear regression model. % was for categorical variables. The *p*-value was calculated by the weighted Chi-square test. The results were statistically significant when *p* < *0.05*

BMD: bone mineral density; CRP: HS C-reactive protein; ALT: alanine aminotransferase; HDL: high-density lipoprotein cholesterol; UHR: serum uric acid-to-high-density lipoprotein cholesterol ratio

### Multiple linear regression and stratified analysis

In Table 2, the multiple linear regression analysis showed a significant positive association between UHR and femoral neck BMD in Model 1 (unadjusted) and Model 2 (adjusted for age, gender, and race), with *p*-values < 0.05 (*β* = 0.0088, *p* < 0.001 in Model 1 and *β* = 0.0052,* p* < 0.001 in Model 2). However, in Model 3 (adjusted for additional covariates), the association was no longer significant (*β* = 0.0021, *p* = 0.196). Stratified analyses by gender showed significant associations in Models 1 and 2 for both males (*β* = 0.0053, *p* < 0.001) and females (*β* = 0.0046, *p* < 0.001), but not in Model 3. Stratification by age revealed significant associations for T1 in Models 1 and 2 (*p* < 0.001), but not for T2 (*p* = 0.927) and T3 (*p* = 0.123) in Model 3. In stratified race analysis, significant associations were observed for Mexican Americans (*p* < 0.001 in Models 1 and 2 and *p* = 0.012, *β* = 0.0145 in Model 3), Other Hispanics (*p* < 0.001 in Models 1 and 2, *p* = 0.445 in Model 3), and Non-Hispanic Whites (*p* < 0.001 in Models 1 and 2, *p* = 0.377 in Model 3). Non-Hispanic Blacks showed a significant result in Model 2 (*p* = 0.009), but no significance in Model 3 (*p* = 0.063). Stratification by physical activity showed no significant associations across all models (*p* > 0.05).

**Table 2:** The association between UHR and femoral neck bone mineral density in multiple linear regression analysis and subgroup analysis

Exposure	Model1 *β* (95%CI), *p*-value	Model2 *β* (95%CI), *p*-value	Model3 *β* (95%CI), *p*-value
UHR	0.0088 (0.0076, 0.0100) < 0.001	0.0052 (0.0040, 0.0065) < 0.001	0.0021 (− 0.0011, 0.0053) 0.196
Stratified by gender			
Male	0.0053 (0.0037, 0.0069) < 0.001	0.0052 (0.0037, 0.0067) < 0.001	− 0.0006(− 0.0053, 0.0042) 0.813
Female	0.0046 (0.0025, 0.0067) < 0.001	0.0054 (0.0034, 0.0074) < 0.001	0.0003 (− 0.0057, 0.0063) 0.923
Stratified by age			
T1	0.0090 (0.0070, 0.0110) < 0.001	0.0053 (0.0030, 0.0077) < 0.001	0.0033 (− 0.0023, 0.0089) 0.252
T2	0.0085 (0.0067, 0.0103) < 0.001	0.0056 (0.0035, 0.0076) < 0.001	− 0.0003(− 0.0056, 0.0051) 0.927
T3	0.0099 (0.0079, 0.0119) < 0.001	0.0053 (0.0032, 0.0074) < 0.001	0.0048 (− 0.0013, 0.0109) 0.123
Stratified by race			
Mexican American	0.0060 (0.0028, 0.0093) < 0.001	0.0025 (− 0.0008, 0.0059) 0.136	0.0145 (0.0033, 0.0257) 0.012
Other Hispanic	0.0086 (0.0055, 0.0118) < 0.001	0.0041 (0.0004, 0.0078) 0.031	0.0045 (− 0.0069, 0.0158) 0.445
Non-Hispanic White	0.0096 (0.0076, 0.0115) < 0.001	0.0064 (0.0043, 0.0085) < 0.001	0.0025 (− 0.0031, 0.0082) 0.377
Non-Hispanic Black	0.0049 (0.0025, 0.0073) < 0.001	0.0032 (0.0008, 0.0056) 0.009	− 0.0058 (− 0.0118, 0.0003) 0.063
Other Race	0.0076 (0.0050, 0.0102) < 0.001	0.0019 (− 0.0007, 0.0045)0.162	0.0003 (− 0.0062, 0.0068) 0.932
Stratified by physcial activity			
Moderate	0.0021 (− 0.0052,0.0095) 0.569	0.0016 (− 0.0049, 0.0082) 0.626	− 0.0025 (− 0.0053, 0.0003) 0.081
Severe	0.0030 (− 0.0013,0.0072) 0.168	− 0.0025 (− 0.0063,0.0012) 0.188	− 0.0011 (− 0.0028, 0.0006) 0.193

Model 1: no covariates were adjusted

Model 2: age, gender, and race were adjusted

Model 3: gender, age, race, education, marital status, family income-to-poverty ratio, body mass index, smoking status, alcohol use, hypertension, diabetes, physical activity, CRP, ALT, albumin, urea nitrogen, creatinine, calcium, phosphorus, uric acid, HDL were adjusted

The subgroup analyses stratified by gender, age, race. A *p*-value < 0.05 indicates that the result is statistically significant

### Smooth curve fitting and threshold effect analysis

**Fig. 2:**
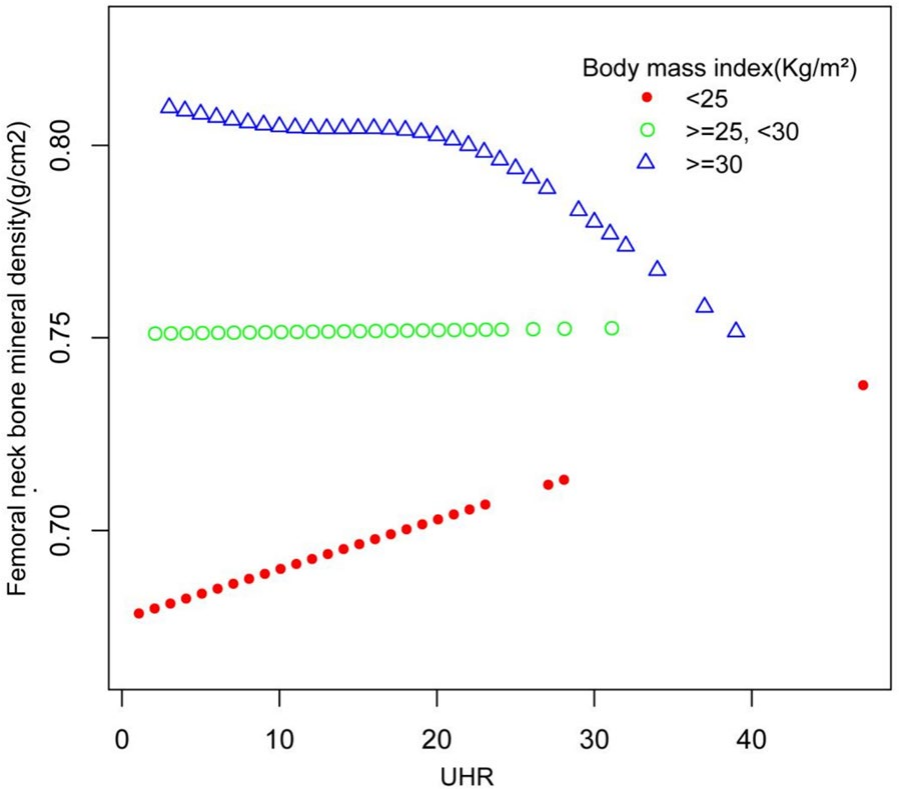
Smooth curve fitting of the association between UHR and femoral neck bone mineral density (g/cm.^2^). Red line: represents the population with a BMI less than 25. Green line: represents the population with a BMI between 25 and 30. Blue line: represents the population with a BMI of 30 or higher. The inflection point appears at UHR 19, indicating a change in the relationship between UHR and bone mineral density (BMD) at that specific value. This figure illustrates the variation in the relationship between UHR and femoral neck BMD across different BMI groups, with a critical threshold observed at UHR 19

The relationship between UHR and BMD was assessed across different BMI categories. A smooth curve analysis identified an inflection point at UHR 19, indicating a change in the association between UHR and BMD (Fig. 2). Using two-piecewise linear regression (Table 3), UHR values below 19 showed a significant positive association with BMD (adjusted *β* = 0.0054, 95% CI 0.0012 to 0.0096,* p* = 0.013), while values above 19 indicated a non-significant negative association (adjusted *β* = − 0.0016, 95% CI − 0.0061 to 0.0029,* p* = 0.478). The two-piecewise linear model demonstrated a better fit than the standard linear model (*p* < 0.05), particularly in detecting the threshold effect at UHR 19.

**Table 3:** Threshold effects of UHR and femoral neck bone mineral density in a middle-aged and elderly population with three classifications of BMI analyzed using two-piecewise linear regression models

Femoral neck bone mineral density	Adjusted *β* (95% CI) *P-value*
Body mass index	
Fitting by the standard linear model	0.0021 (− 0.0011, 0.0053) 0.196
Fitting by the two-piecewise linear model	
Inflection point	19
UHR < 19	0.0054 (0.0012, 0.0096) 0.013
UHR > 19	− 0.0016 (− 0.0061, 0.0029) 0.478
Log likelihood ratio	0.020

Log likelihood ratio is statistically significant (*p* < 0.05), which indicates that the two-piecewise linear model fits the data better than the standard linear model, especially in terms of identifying the threshold effect at a UHR of 19

## Discussion

This study examined the link between UHR and BMD across different BMI categories, revealing a non-linear relationship. An inflection point was identified at UHR 19. Below this threshold, UHR showed a significant positive association with BMD (*β* = 0.0054, *p* = 0.0127), while above 19, the association became negative but was not statistically significant (*β* = − 0.0016, *p* = 0.4784). These findings indicate that UHR’s influence on BMD may vary with BMI and metabolic factors, highlighting its potential as a nuanced biomarker for assessing bone health.

Several studies have examined the relationship between uric acid, cholesterol, and BMD using NHANES data. For example, Xie et al. found a positive association between HDL cholesterol and BMD in U.S. adults, suggesting that higher HDL levels contribute to better bone health [17]. However, few studies have specifically focused on the uric acid to HDL cholesterol ratio (UHR) and its impact on BMD. Although previous studies have shown that HDL-C plays a positive role in bone metabolism, our research further emphasizes the impact of UHR on BMD and explores the role of the critical threshold at UHR 19. In contrast, a study by J Wang et al. suggested a potential inverse relationship between UHR and lumbar spine BMD [25], adding complexity to the understanding of UHR’s role in bone health. Similarly, the study by Liu et al. also observed a positive association between UHR and femoral neck BMD in older adults [26], further emphasizing the importance of studying weight-bearing bones, such as the femoral neck, which are more sensitive to metabolic changes [27]. The observed threshold effect at UHR 19 may represent a critical point beyond which the protective effects of uric acid, through its antioxidant properties, are offset by pro-inflammatory processes [28, 29].

The underlying mechanisms behind the association between UHR and BMD may involve multiple pathways. Uric acid is known to act as both an antioxidant and a pro-oxidant under different conditions [30–32]. At lower levels, it may reduce oxidative stress, which is beneficial for bone formation by enhancing osteoblast activity and inhibiting osteoclast-induced bone resorption [33]. However, when uric acid levels rise, it triggers inflammatory responses through pathways including NOD-, LRR-, and pyrin domain-containing protein 3 (NLRP3) inflammasome activation, which induces Interleukin-1 beta (IL-1β) release and stimulates Tumor necrosis factor alpha (TNF-α) and Interleukin-6 (IL-6) production. Uric acid also activates mitogen-activated protein kinase (MAPK) and nuclear factor kappa-light-chain-enhancer of activated B cells (NF-κB) signaling pathways, driving further transcription of pro-inflammatory cytokines and increasing oxidative stress via reactive oxygen species, which reinforce NF-κB and MAPK activity [34–37]. This collective inflammatory response promotes osteoclast activity, accelerating bone degradation [34]. Additionally, HDL cholesterol influences bone metabolism by activating the Wnt/β-catenin signaling pathway, a key regulator of osteoblast differentiation [38]. The Wnt/β-catenin signaling pathway is essential in regulating osteoblast differentiation. It enhances Runt-related transcription factor 2 (RUNX2) expression by facilitating β-catenin’s accumulation in the nucleus, where it binds T-cell factor/lymphoid enhancer factor (TCF/LEF) transcription factors to activate RUNX2. Known as the master regulator of osteoblast differentiation, RUNX2 initiates gene expression necessary for bone formation and mineralization. Acting downstream, SP7 transcription factor (Osterix) is critical for the final maturation of osteoblasts, binding to promoters of osteogenic genes like osteocalcin and bone sialoprotein, which are crucial for mineralization. RUNX2 activation also promotes Osterix expression, establishing a positive regulatory loop supported by Wnt/β-catenin signaling, ensuring coordinated osteoblast differentiation and maturation [39–42]. In our study, the positive correlation between UHR and BMD below the threshold suggests that HDL-C exerts a dominant protective effect at lower uric acid levels. Once uric acid levels rise above 19, its inflammatory effects may outweigh the protective role of HDL-C.

This study has several strengths. First, it uses data from NHANES, a large, nationally representative dataset, which enhances the generalizability of the findings to the U.S. population. Second, the use of a two-piecewise linear regression model allowed us to detect a non-linear relationship between UHR and BMD, which was overlooked in previous studies that employed standard linear models. Additionally, stratified analyses by BMI, gender, and ethnicity provided more nuanced insights into the metabolic factors affecting different subpopulations, revealing a significant association among Mexican Americans even after controlling for covariates. A study on older non-Hispanic Caucasian and Mexican–American women found lean mass strongly supports bone density, while fat mass has a weaker effect [43]. This suggests that balanced lean mass, uric acid, and HDL-C are crucial for bone health across these groups. However, the study also has limitations. The cross-sectional nature of the NHANES dataset precludes causal inferences, and longitudinal studies are needed to confirm whether changes in UHR directly impact BMD over time. Moreover, although we adjusted for multiple covariates, residual confounding from unmeasured factors such as diet, physical activity, and genetic predispositions cannot be ruled out. Finally, the reliance on a single measure of BMD (the femoral neck) may limit the generalizability of the findings to other skeletal sites, such as the lumbar spine [44, 45], which was not assessed in this study.

## Conclusion

This study demonstrates a non-linear relationship between UHR and BMD, with a crucial threshold at UHR 19. Below this point, UHR is positively correlated with BMD, while above it, the relationship becomes negative. These results indicate that UHR could serve as a valuable biomarker for evaluating bone health, especially in individuals at metabolic risk. Future longitudinal research is necessary to confirm these findings and explore potential interventions targeting UHR to improve bone health outcomes in specific populations.

## Data Availability

No datasets were generated or analysed during the current study.

## Abbreviation

BMD: Bone mineral density
HDL-C: High-density lipoprotein cholesterol
UHR: Uric acid to HDL cholesterol ratio
NHANES: National Health and Nutrition Examination Survey
DXA: Dual-energy X-ray absorptiometry
UA: Uric acid
BMI: Body mass index
CRP: C-reactive protein
ALT: Alanine aminotransferase
NLRP3: NOD-, LRR-, and pyrin domain-containing protein 3
IL-1β: Interleukin-1 beta
TNF-α: Tumor necrosis factor alpha
IL-6: Interleukin-6
NF-**κ**B: Nuclear factor kappa-light-chain-enhancer of activated B cells
MAPK: Mitogen-activated protein kinase
Wnt/β-catenin signaling pathway: Wingless-related integration site/beta-catenin signaling pathway
TCF/LEF: T-cell factor/lymphoid enhancer factor
RUNX2: Runt-related transcription factor 2
Osterix: SP7 transcription factor
